# Cutaneous sarcomatoid squamous cell carcinoma of the scalp: a case report

**DOI:** 10.1093/jscr/rjaf456

**Published:** 2025-07-01

**Authors:** Agnes Nampijja, Jackson Kakooza, Catherine Lewis, William M Mutumba

**Affiliations:** Department of Surgery, St. Joseph’s Hospital Kitovu, PO Box 524, Masaka, Uganda; Department of Surgery, St. Joseph’s Hospital Kitovu, PO Box 524, Masaka, Uganda; Department of Surgery, St. Joseph’s Hospital Kitovu, PO Box 524, Masaka, Uganda; Department of Surgery, Quillen College of Medicine, East Tennessee State University, PO Box 70575, Johnson City, TN 37614, United States; Lancet Laboratories, Nakasero Hill, Plot 1, Kyadondo Road, Kampala, Uganda

**Keywords:** sarcomatoid carcinoma, sarcomatoid squamous cell carcinoma, cutaneous tumor, spindle cells

## Abstract

Sarcomatoid squamous cell carcinoma (SSCC) is a malignant tumor with epithelial and spindle cell components. It is common in visceral organs and rarely presents as a cutaneous tumor. We present a case of a 55-year-old Black male with multiple recurrences of SSCC. Prompt diagnosis and complete excision is prudent to prevent recurrence or further spread to adjacent structures.

## Introduction

Sarcomatoid carcinoma is a rare histologic subtype characterized as a cancer of unknown primary (CUP). It is defined as a poorly differentiated carcinoma with sarcoma-like differentiation and a component of spindle cells [1]. Cutaneous squamous cell carcinoma (cSCC) is the second most prominent form of skin malignancy, occurring frequently in older men with fair complexion and extensive sun exposure [2]. Sarcomatoid squamous cell carcinoma (SSCC), a variant of SCC, is a biphasic tumor with malignant epithelial and spindle cell components [3, 4].

Most cases of SSCC occur in the visceral organs [3, 5]. Other common locations include the oral cavity and oropharynx, but they have also been reported to occur anywhere in the head and neck region [6]. Cutaneous SSCC is a rare form of SSCC with unknown causes and clinical manifestations that are dependent on the location. SSCC rarely develops as a primary cutaneous tumor [3], and there are very few cases of cutaneous SSCC reported in the English literature [5, 7]. We report a case of a malignant cutaneous tumor with sarcomatoid differentiation in a 55-year-old Black male.

## Case presentation

A 55-year-old Black male, with a past medical history of hypertension, presented with a recurrent forehead swelling. He was HIV seronegative. The swelling initially started ten years prior. A computed tomography (CT) scan of the head at that time demonstrated a forehead mass with no cranial involvement. The mass was excised completely. The mass recurred the following year. A fine needle aspiration biopsy was performed with histology indicating a benign cystic mass. Excision was performed again but the mass reoccurred and was re-excised a third time. All the surgeries above were done at different health centers.

**Figure 1:**
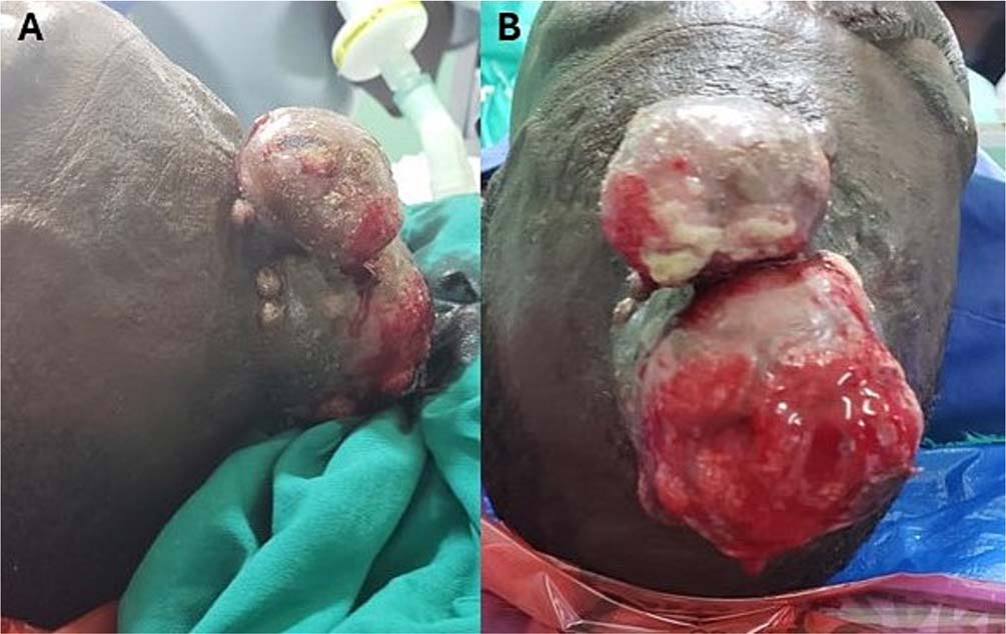
(A) Bicornuate, ulcerated forehead mass with irregular borders. (B) The mass was friable with areas of bleeding and sloughing.

**Figure 2:**
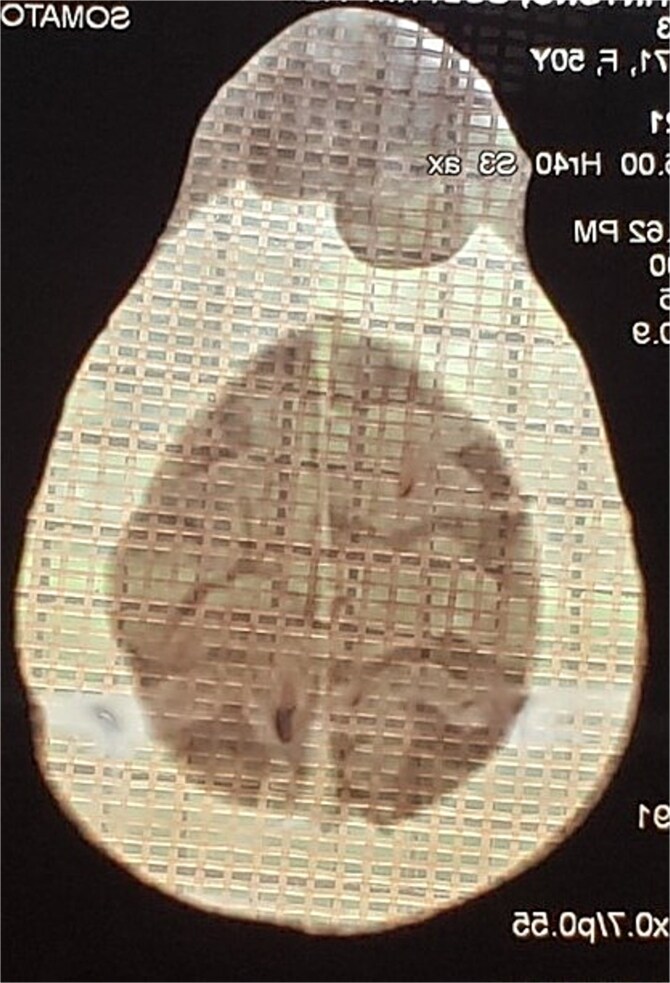
CT scan of the head demonstrating a frontal forehead mass with extension to the underlying skull.

He presented to our facility with a 1-week history of spontaneous bleeding from a recurrent forehead swelling. There were associated palpitations, malaise, fever, anorexia, dizziness, and throbbing headaches. There was no altered mentation, loss of consciousness, or convulsions. On physical examination, he was alert, ill-appearing, and cachectic. He was afebrile with a pulse rate of 104 bpm and a blood pressure of 140/80 mmHg. There was moderate pallor with no lymphadenopathy, no clubbing, and no edema. There was a foul smelling, bicornuate, ulcerated forehead mass measuring 10 × 8 × 6 cm, with irregular borders. The mass was pink and friable with bleeding and areas of sloughing. It was soft with a smooth surface and no temperature gradient. There was tenderness to palpation with extension to the underlying bone (Fig. 1). A CT scan of the head was performed that demonstrated a frontal scalp mass with underlying invasion of the bone (Fig. 2).

**Figure 3:**
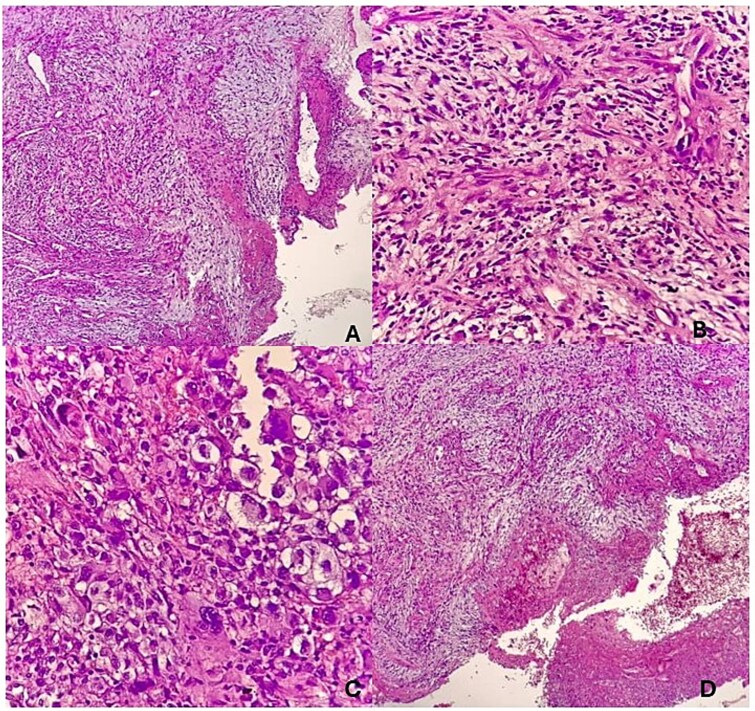
(A) Sections show tissue with an ulcerated squamous cell epithelium, having a false membrane and an acute on chronic inflammatory cellular infiltrate. (B) The stroma has a fibromyxoid component with aypical spindle and fat cells. (C) The cells are pleomorphic with prominent nucleoli. (D) There is increased vascularization.

The patient was taken to the operating room for biopsy. Histological analysis demonstrated sections of tissue with an ulcerated squamous cell epithelium, having a false membrane and an acute on chronic inflammatory cellular infiltrate. The stroma had a fibromyxoid component with atypical spindle and fat cells with increased vascularization. Findings were consistent with a tumor with sarcomatoid differentiation and SSCC (Fig. 3). Immunohistochemical staining was advised for further tumor typing but was not performed due to financial constraints. The patient was subsequently lost to follow-up.

## Discussion

Sarcomatoid carcinoma is a rare variant of SCC encompassing 3% of all SCCs of the head and neck region [8]. They are more common in males, predominantly arise in visceral organs, and rarely present as primary cutaneous tumors [9, 10], with ˂150 cases reported in the literature [11]. Risk factors for cutaneous involvement include immunosuppression, previous irradiation, and smoking and/or alcohol consumption. Prolonged sun exposure and effects of toxins are also risk factors for skin involvement [10, 12]. Our patient did not use alcohol and had no previous history of radiation.

The diagnosis of SSCC is primarily done by histological analysis and in some cases immunohistochemistry. Immunohistochemical analysis shows biphasic morphology consisting of spindle-shaped neoplastic cells along with epithelioid cells. Mixtures of SCC with sarcomatoid spindle cells, basal cell carcinoma, and true mesenchymal sarcoma are combinations of the biphasic morphology that may exist [3]. Classic SCC histology is typically seen merging with a spindle cell component [12].

Immunohistochemical staining can also be useful as an indicator of epithelial differentiation as spindle cells in sarcomatoid carcinoma may be present without clear epithelial differentiation, making the delineation between sarcomatoid carcinoma and primary sarcoma difficult [3]. Positivity for cytokeratin and p63 characterizes the SCC component of SSCC, while the sarcomatous region will demonstrate positivity for p63 and vimentin [1, 3, 4]. Immunohistochemistry was not performed in our case due to financial restraints.

Complete excision of all affected areas is standard treatment for cutaneous SSCC, particularly if satellite lesions are present [3, 4]. Complete surgical excision with a 10 mm circumferential margin, including the underlying fascia, is suggested [3, 5]. The role of radiation is controversial as radiation has been shown to be a risk factor for sarcomatoid carcinoma [12, 13]. Radiation therapy may be considered an option for inoperable patients or for those in which the surgical margins are positive or when there are nodal metastases at the time of diagnosis [13].

Tumors present for ˃3 years, tumor size ˃2 cm, recent tumor growth, regional lymph node metastasis, adnexal differentiation, and patient age ˂65 years are poor prognostic factors for sarcomatoid carcinoma [3]. In this case, our patient was a 55-year-old male with a long-standing tumor measuring at least 10 cm, making him a high-risk patient.

## Conclusion

SSCC is rare, and its risk factors are multiple and diverse. Its diagnosis is tricky and expensive, especially in a low-income country. Early diagnosis is key to preventing metastasis and recurrence. Complete surgical excision of the local tumor is crucial in the management and prevention of recurrence.

## Data Availability

The data that support the findings of this case report are available from the corresponding author upon reasonable request.
